# Epidemiological, Phylogenetic, and Resistance Heterogeneity Among *Acinetobacter baumannii* in a Large U.S. Deep South Healthcare system

**DOI:** 10.1093/ofid/ofae458

**Published:** 2024-08-10

**Authors:** Emma Graffice, Derek B Moates, Sixto M Leal, Megan Amerson-Brown, Juan J Calix

**Affiliations:** Department of Medicine, Division of Infectious Disease, University of Alabama at Birmingham, Birmingham, Alabama, USA; Department of Pathology, Division of Laboratory Science, University of Alabama at Birmingham, Birmingham, Alabama, USA; Department of Pathology, Division of Laboratory Science, University of Alabama at Birmingham, Birmingham, Alabama, USA; Department of Pathology, Division of Laboratory Science, University of Alabama at Birmingham, Birmingham, Alabama, USA; Department of Medicine, Division of Infectious Disease, University of Alabama at Birmingham, Birmingham, Alabama, USA

**Keywords:** carbapenem-resistant Acinetobacter baumannii, molecular epidemiology, multidrug resistance, heteroresistance, medical microbiology

## Abstract

**Background:**

*Acinetobacter baumannii* (*Ab*) disease in the United States is commonly attributed to outbreaks of 1 or 2 monophyletic carbapenem resistance (CR) *Ab* lineages that vary by region. However, there is limited knowledge regarding CR*Ab* epidemiology and population structures in the U.S. Deep South, and few studies compare contemporary CR and carbapenem-susceptible (Cs) *Ab*, despite relative prevalence of the latter.

**Methods:**

We performed a multiyear analysis of 2462 *Ab* cases in a large healthcare system in Birmingham, AL, and 89 post-2021 *Ab* isolates were sequenced and phenotyped by antibiotic susceptibility tests.

**Results:**

Although the cumulative CR rate was 17.7% in our cohort, rates regularly increased in winter months as result of seasonal changes in case incidence of Cs*Ab*, specifically. Genotyped CR*Ab* belonged to clonal group (CG) 1, CG2, CG108, CG250, or CG499, with local clones of CG108, CG250, and CG499 persisting over multiple months. There was no clonal expansion of any Cs*Ab* lineage. Among CR*Ab* isolates, levels of β-lactam antibiotic resistance and the repertoire of related genetic resistance determinants, which included the novel CR-conferring FtsI A515V polymorphism, differed according to CG. CG108 and CG499 isolates displayed specific heteroresistance to sulbactam and trimethoprim/sulfamethoxazole, respectively, which resulted in discrepant susceptibility results in microbroth versus agar-based antibiotic susceptibility tests modalities.

**Conclusions:**

We report an unusually high degree of CR*Ab* phylogenetic diversity principally driven by emergent U.S. lineages harboring novel resistance elements that must be incorporated into diagnostic, surveillance, and preclinical research efforts.

Carbapenem-resistant (CR) *Acinetobacter baumannii* (*Ab*) is considered a top priority for research by the World Health Organization [1] and U.S. Centers for Disease Control and Prevention [2]. This incidental pathogen is able to survive in various environments and regularly displays multidrug-resistant (MDR) phenotypes mediated by intrinsic *Acinetobacter*-derived cephalosporinase (ADC) class C β-lactamases and OXA class D β-lactamases, de novo polymorphisms in antibiotic targets, and a variety of extrinsic resistance genes—including OXA and non-OXA carbapenemases—acquired via mobile genetic elements (eg, plasmids, transposons). CR*Ab* disease is conventionally linked to hospital-acquired (HA) respiratory, endovascular, wound, and urinary infections mainly attributable to a subset of MDR global lineages within the otherwise phylogenetically diverse species [3–5].

*Ab* strains are routinely genotyped according to 1 of 2 multilocus sequence type (MLST) schemes [6], and here we exclusively use the Pasteur MLST scheme [7]. Single locus variant sequence types can be further grouped into monophyletic lineages, or clonal groups (CGs), often named according to the prevailing sequence type [8]. CR*Ab*-associated lineages CG2, CG1 and CG79 (the latter also known as Clade E [9]) comprised 61%, 5%, and 3%, respectively, of the ∼3500 genomes available on NCBI in early 2019 [10]. CG2 comprised 85% of U.S. CR*Ab* isolates in the first nationwide survey performed between 2008 and 2009 [11] and 64% of isolates from Centers for Disease Control and Prevention sentinel sites between 2013 and 2017 [12]. In contrast, CG2 comprises a small portion of sequenced isolates from Latin American countries [8, 10] and U.S. single-center studies reveal significant variability between regions and over time [9, 12–15]. For example, most pre-2012 CR*Ab* isolates in a St. Louis, Missouri, healthcare system were CG2 or CG79 [11], but local clones of the unrelated ST406 (CG406, also known as USA-clone-1 [16]) and ST499 (CG499), the latter of which was first detected in Chicago in 2010 [17], rapidly became the prevailing CR*Ab* lineages between 2017 and 2019 [14].

Awareness of *Ab* CGs propagating in a region could inform clinical practice because recent evidence suggests that CR*Ab* lineages each display characteristic antibiotic susceptibility patterns and may differ in their epidemiological behavior [8, 14]. Notably, little is known regarding the population structure of *Ab* in the U.S. Deep South. Other than the inclusion of 6 Florida isolates [11] and a few Louisiana isolates reported during the writing of this manuscript [8], surveys have not included isolates from Deep South/Southeast region (including Tennessee, Mississippi, Georgia, and Alabama), where unique climate socioeconomic factors may selectively favor traits absent from pools of this environmental microbe in other regions. In addition, most recent surveys exclude cases associated with carbapenem-susceptible (Cs) *Ab,* despite these being a common cause of *Ab* disease and more representative of the heterogeneity within the species [14].

Here, we report findings from an ongoing investigation of *Ab* population dynamics in the U.S. Deep South. We compare the epidemiology of Cs*Ab* and CR*Ab* isolates identified over 12 years in a large healthcare system in Birmingham, Alabama, and characterize the current population structure of clinically relevant *Ab* by isolate whole-genome sequencing. We report a link between emergent lineages and atypical antimicrobial resistance phenotypes and contextualize findings within the greater *Ab* population structure in the United States.

## MATERIALS AND METHODS

### Study Location and Period

This study was approved by the University of Alabama at Birmingham (UAB) institutional review board (# 30003572 and 30008212) and was performed in the UAB Healthcare system from 1 July 2010 to 31 December 2022. UAB is the largest integrated inpatient and outpatient system serving Birmingham, Alabama, and surrounding areas. UAB Hospital and affiliated clinics use a central UAB Clinical Microbiology Laboratory (UAB-CML) on the main campus, which performed species identification of bacterial isolates by automated biochemical methods or matrix-assisted laser desorption/ionization and time-of-flight mass spectroscopy. Per routine protocol, the UAB-CML tests all *Acinetobacter* isolates via microbroth dilution antimicrobial susceptibility testing (MB-AST) using the Beckman Coulter Microscan WalkAway Plus AST system, against the following antibiotics: ampicillin-sulbactam (SAM), cefepime (FEP), ceftazidime (CAZ), ciprofloxacin, gentamicin, levofloxacin, trimethoprim-sulfamethoxazole (SXT), tobramycin, imipenem (IPM), and meropenem (MEM). AST and susceptibility reporting is performed according to Clinical and Laboratory Standards Institute (CLSI) guidelines [18].

### Retrospective Study Design and Definitions

The UAB Cerner electronic medical record systems was queried using the Informatics for Integrating Biology and the Bedside tool hosted by the UAB Center for Clinical and Translation Science to identify cases and retrieve patient demographics, culture tissue source, hospital day of culture (if applicable), and AST interpretation results. Only first culture per patient (“index culture”) identified over the course of routine care and containing the following was eligible for inclusion: “*Acinetobacter baumannii*” (n = 419), “*Acinetobacter baumannii* complex” (n = 651), “*Acinetobacter baumannii* complex/*haemolyticus*” (n = 5), or “*Acinetobacter baumannii*/*haemolyticus*” (n = 1387). Isolates reported as “intermediate” or “resistant” to either IPM or MEM were defined as “carbapenem nonsusceptible *Ab* (CNS*Ab*). The CLSI *Acinetobacter* spp AST breakpoints for IPM and MEM were updated in 2014. Minimum inhibitory concentration (MIC) values were not retrievable in our retrospective cohort, so we relied on the carbapenem susceptibility interpretations according to breakpoints at the time of testing. A total of 169 of 186 (90.8%) pre-2015 CNS*Ab* index isolates were reported “resistant” to at least 1 carbapenem, and, thus, meet CNS*Ab* criteria using updated breakpoints. Cases were classified into 5 anatomical categories according to specimen source: “respiratory,” “skin and soft tissue/musculoskeletal” (SST/MSK), “urinary,” “blood” (including central lines, endovascular devices, or grafts), or “other.” Cases were defined as HA if the index culture was performed ≥48 hours from time of hospitalization and before discharge. All other cases were defined as nHA. To evaluate seasonal trends, cases in 2011–2022 were grouped into 4 quarters according to the month index culture was obtained: 1 (January–March), 2 (April–June), 3 (July–September), and 4 (October–December).

### Prospective Isolate Banking

Since November 2021, clinical isolates typed as an *Acinetobacter* species by matrix-assisted laser desorption/ionization and time-of-flight mass spectroscopy in the UAB-CML were identified via biweekly electronic medical record queries and were eligible for inclusion. For each colony morphotype identified as *Acinetobacter* on culture plates, 2–5 bacterial colonies were subcloned and transferred to the research laboratory for processing and storage. For this study, we arbitrarily chose 89 presumptive *Ab* isolates identified between 1 November 2021 and 31 November 2022 for sequencing and susceptibility testing analysis (Supplementary Data 1). Here, study isolates are named by number (ie, isolate 002 denotes UAB_Ab002, etc.). *Ab* isolates were not routinely banked at UAB before this study.

### Whole-Genome Sequencing, Assembly and Comparative Genomics

A full description of the well-established processing pipelines [14] used for genome assembly and comparative genomics analyses is provided in the Supplementary Text. The earliest isolate with an MLST and/or antimicrobial resistance gene profile per person, was denoted as an index isolate and subsequent homologous isolates denoted as non-index isolates.

### Kirby-Bauer AST

Study isolates were tested by Kirby-Bauer disk diffusion AST (KB-AST) in our research laboratory with Mueller-Hinton agar against SAM, FEP, CAZ, MEM, IPM, SXT, ciprofloxacin, gentamicin, and doxycycline. Zone of clearance breakpoints were determined and interpreted according to CLSI guidelines [18], and isolates “resistant” to MEM or IPM were designated “CR*Ab*”. In cases in which distinct colonies were repeatedly observed growing up to the border of the antibiotic disk but readily distinguishable from the lawn's major border, we subcloned bacteria from colonies at the disk border (“disk subclone”) or from the main lawn (“lawn subclone”). Subclones were plated and incubated at 37°C for 16 hours on LB Miller agar without antibiotics and retested by KB-AST the next day. Plate images were acquired from a height of 6.3 inches using an iPhone 14 Pro Max with 1× lens and arranged using Illustrator (Adobe, USA).

### Global Analysis With Genomes From Other Contemporary U.S. *Ab* Isolates

Full description of the genome identification and comparative genomic meta-analysis is provided in the Supplementary Text. Briefly, an NCBI database query performed on 18 September 2023 identified 774 whole genome sequences of *Ab* isolated in the United States since 1 January 2010 per peer-reviewed reports. We performed de novo genome assembly using NCBI SRA files and, when available, extracted epidemiological and microbiological metadata (Supplementary Data 2). Genomes were binned into CGs according to MLST analysis, and comparative genomic analysis was done within each CG. In the case where a CG of interest was absent among these binned genomes (ie, CG108), we queried NCBI for homologous genomes whose metadata indicated they were from post-2009 U.S. isolates.

### Statistical Analyses

Univariate analyses were performed with SPSS v29 (IBM, USA) or RStudio software v 4.1.2 [19]. Chi-squared or odds ratio test were performed for comparing categorical variables. Mann-Whitney test with Bonferroni adjustment for multiple comparisons was performed for continuous variables. Statistical significance was defined as *P* <.05.

## Supplementary Material

ofae458_Supplementary_Data

## Figures and Tables

**Figure 1.**
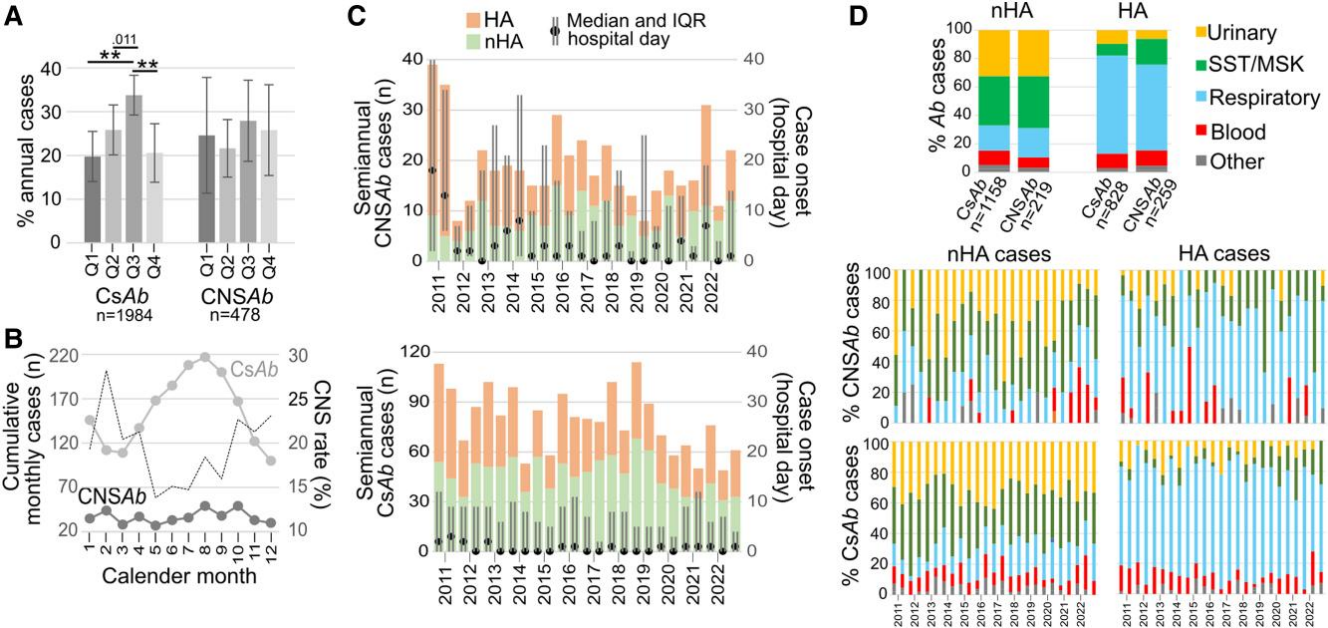
Divergent epidemiological features of *Ab* cases at UAB, July 2010-December 2022. *A*, Proportion of annual carbapenem-susceptible *A. baumannii* (CsA*b*) and carbapenem nonsusceptible Ab (CNS*Ab*) cases occurring in each calendar quarter (Q1-Q4) during 2011–2022. **< .001. *B*, Cumulative case total for each month (left y-axis) of Cs*Ab* and CNS*Ab* (light and dark gray, respectively) and monthly CNS rate (dotted line, right y-axis) among cases from 2011 to 2022. *C*, Semiannual totals (left y-axis) of hospital-acquired (HA) and non–hospital acquired (nHA) CNS*Ab* (top) and Cs*Ab* (botom) cases. Lines and error bars depict the median and interquartile range (IQR) for hospital day of isolation among cases in each period (right y-axis). *D*, Cumulative (top) and semiannual (bottom) percentage of CNS*Ab* and Cs*Ab* index isolates from each tissue source, separated by nHA (left) and HA (right) cases.

**Figure 2.**
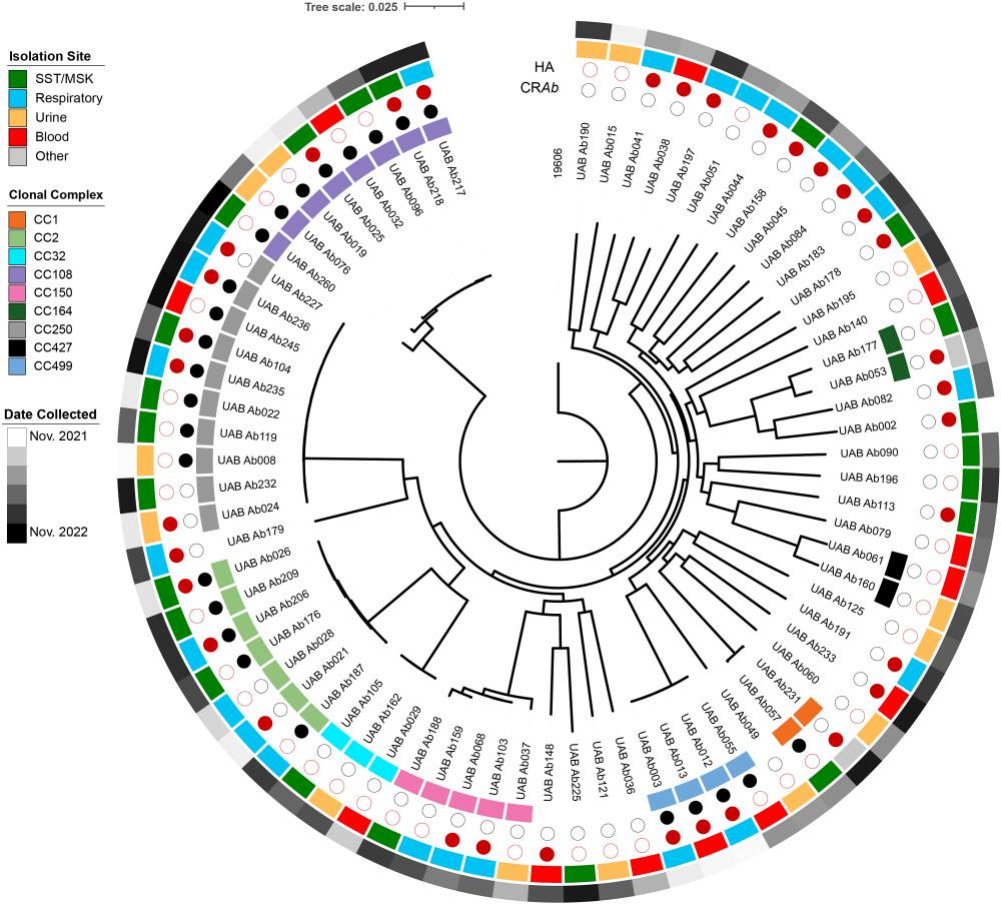
Diversity of *Ab* isolates at UAB hospitals, 2021–2022. Maximum likelihood phylogenetic tree of UAB Ab index isolates according to alignment of 2072 core genes, rooted to the genome of reference strains 19 606 (labeled). Inner label ring denotes CG with more >1 index isolate. Filled circles denote CRAb and isolates associated with HA cases. Outer rings represent isolate metadata according to the corresponding keys, as listed in Supplementary Data 1.

**Figure 3.**
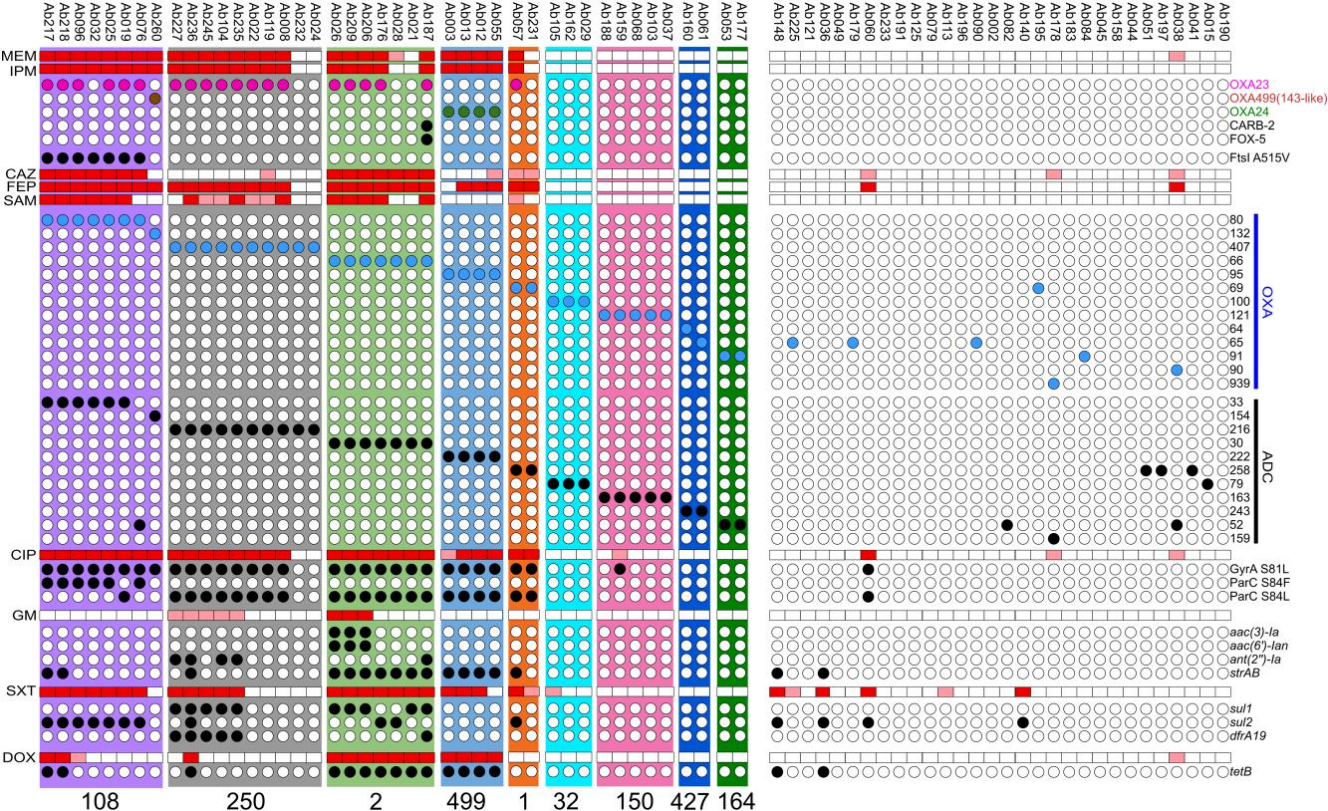
Antibiotic susceptibility and resistance genetic element content of UAB index isolates (top). Tested antibiotics are listed on left and grouped by antibiotic class. Results are presented as “resistant” (dark red), “intermediate” (light pink), or “susceptible” (white) according to CLSI guideline interpretation of Kirby-Bauer results. Circles below each AST group denotes presence (fill circle) or absence of associated resistance genetic elements listed on right. Intrinsic OXA and ADC alleles are listed by allele number. For clarity, only genetic elements present in isolates belonging to multi-isolate CGs (boxed columns with labels below) are included. Results for all UAB isolates and genetic elements are listed in Supplementary Data 4.

**Figure 4.**
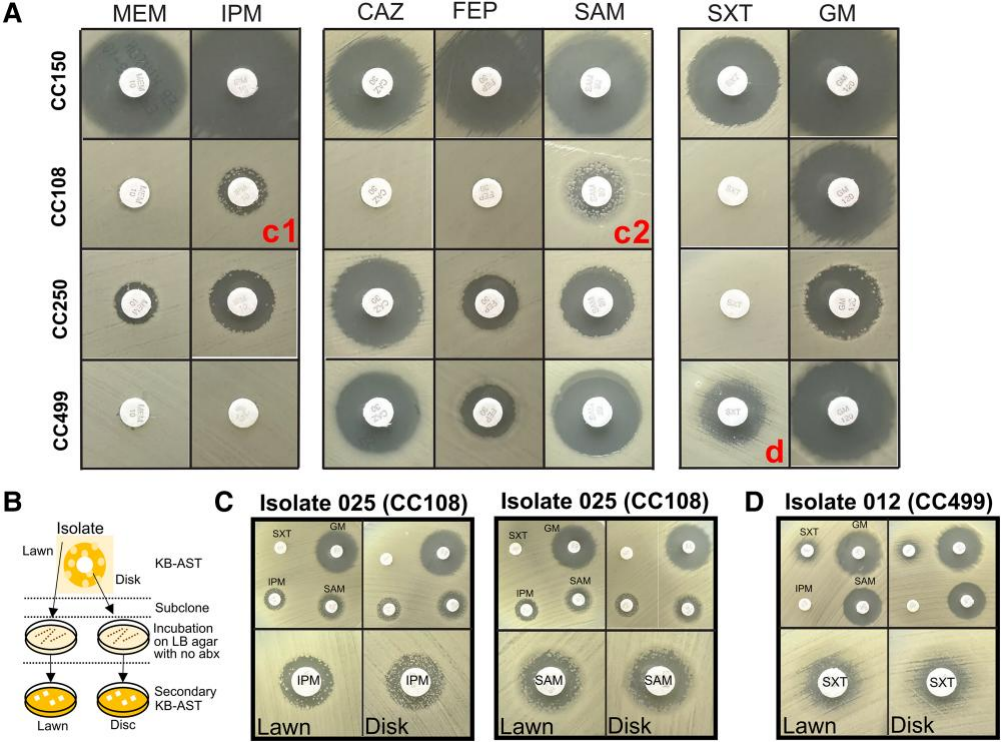
KB-AST identifies susceptibility-variable subpopulations associated with CR*Ab* lineage and antibiotic type. *A*, Representative pictures of the most commonly observed KB-AST results for main CGs in our cohort (left), against different antibiotics (top). Red letters denote samples that were subcloned and incubated overnight on LB agar with no antibiotic followed by secondary KB-AST, as depicted in panel B. *C-D*) Secondary KB-AST of “lawn” (left) and “disk” (right) subclones. Photos show results from multiple antibiotics (top) and magnification of zones of clearance around antibiotics of interest (bottom). Abbreviations: CAZ, ceftazidime; FEP, cefepime; GM, gentamicin; IPM, imipenem; MEM, meropenem; SAM, ampicillin-sulbactam; SXT, trimethoprim/sulfamethoxazole.

**Figure 5.**
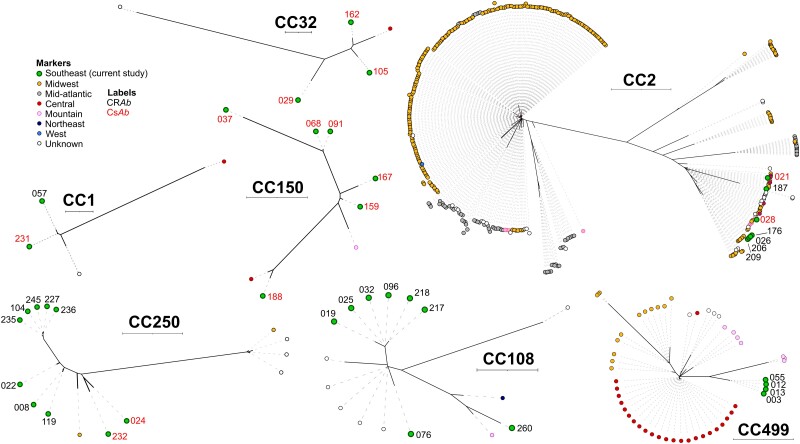
Intra-CG phylogeny of U.S. *Ab* populations identified since 2010. Unrooted maximum likelihood phylogenetic trees drawn according to alignment of core genes shared by isolates within each CG. Tree scales located under CG labels represent to 0.001 substitutions per site, with CG1 and CG32 trees presented as half scale compared to others. Leaf markers represent the regions where each isolate was identified, per the inset key. Numbered labels denote study number of carbapenem-resistant (black) or -susceptible (red) isolates in the UAB study.

## Data Availability

All raw sequence files and assemblies derived from UAB study strains are available under NCBI BioProject PRJNA1005294 (see Supplementary Data 1).

## RESULTS

### Retrospective Analysis Reveals Diverse Clinical Presentations of *Ab* Cases at UAB

We identified 2462 cases associated with *Ab* index cultures between July 2010 and December 2022. A total of 478 cases (19.4%) contained CNS*Ab,* of which 438 (17.7% of total *Ab*) tested “resistant” to MEM and/or IPM. The occurrence of Cs*Ab,* but not CNS*Ab,* cases was higher in third calendar quarters (Figure 1*A*), and the resulting seasonal increase in Cs:CNS ratio led to an increased susceptibility rates among total *Ab* cases between May and October (Figure 1*B*). CNS*Ab* cases were more likely than Cs*Ab* to be HA (54.2% [259/478] vs 41.6% [826/1984], odds ratio [95% confidence interval] = 1.66 [1.36–2.03]). However, HA rates among CNS*Ab* decreased from 76.9% and 85.7% in late 2010 and early 2011, respectively, to 27.4% and 45.5% in both halves of 2022 (Supplementary Data 3). This trend coincided with later CNS*Ab* cases being identified earlier during hospitalization, whereas Cs*Ab* HA rates and median hospital day of isolation were similar in all study periods (Figure 1*C*). Cs*Ab* and CNS*Ab* displayed comparable distribution of isolate tissue sources when stratified according to onset (Figure 1*D*, top) (ie, respiratory isolates predominated among HA cases, while urinary and SST/MSK isolates predominated among nHA cases in nearly all study periods) (Figure 1*D*, bottom). Last, average patient age did not differ between Cs*Ab* (49.0 years) and CNS*Ab* (50.3 years, *P* = .178) cases, but CNS*Ab* was more likely to be isolated from males (odds ratio [95% confidence interval] = 1.49 [1.21–1.84]). Females comprised less than 40% of Cs*Ab* respiratory cases (37.2%) and CNS*Ab* respiratory (37.7%), SST/MSK (33.1%) urinary (34.7%), and other (31.6%) cases (Supplementary Table 1). In summary, CNS*Ab* and Cs*Ab* were both implicated in various case types at UAB, albeit with differential features regarding seasonality, timing of index culture, and patient sex distribution.

### 2021–2022 UAB *Ab* Isolates Demonstrate Phylogenetic Heterogeneity

Genotyping of 89 *Ab* isolates identified in 2021–2022 revealed 42 STs (13 STs newly assigned in this study) distributed among 39 CGs (Supplementary Table 2). Ten case patients had multiple isolates included in our whole-genome sequencing analysis, but only 3 had 2 index isolates of distinct genotypes (Supplementary Figure 1). Thus, our cohort consisted of 73 index isolates from 68 individuals (Figure 2, Supplementary Data 1). A total of 58.9% (n = 43) of index isolates belonged to 9 CGs, each containing ≥2 index isolates obtained from independent cases occurring ≥30 days apart (Figure 2, Supplementary Table 2). Of these, CG108, CG164 and CG427 each contained isolates of 2 STs. The remaining index isolates (n = 30) each belonged to unrelated STs (Supplementary Data 4).

### Antimicrobial Susceptibility was Linked to Lineage-associated and -independent Genetic Elements

We confirmed our cohort was comprised of 26 CR*Ab* and 47 Cs*Ab* index isolates (Figure 3, Supplementary Data 1). CG108 and CG499 exclusively comprised CR*Ab*, CG150, CG164, CG32, and CG427 comprised exclusively Cs*Ab*, and CG1, CG2, and CG250 comprised both. All remaining isolates were Cs*Ab*. ARG analysis revealed all CG499 isolates encode OXA-24, the CG108 isolate 260 encodes OXA-499 (an OXA-143-like carbapenemase), and OXA-23 is encoded in nearly all other CR isolates except isolate 032 (Figure 3). Along with 6 other CG108 isolates, isolate 032 encodes the FtsI A515V polymorphism, a recently described penicillin-binding protein 3 mutation predicted to decrease carbapenem binding [20]. Altogether, carbapenem resistance wholly correlated with the presence of attributable genetic elements (Figure 3, Supplementary Data 4).

The identity of intrinsic OXA and ADC alleles was largely conserved within a lineage and rarely shared between lineages (Figure 3). Independent of OXA-23 presence, CG1 and CG2 isolates displayed greater FEP and CAZ resistance than other Cs*Ab*, including the CG250 Cs*Ab* isolates (Supplementary Figure 2). Conversely, CG250 and CG499 CR*Ab* isolates displayed CAZ susceptibility indistinguishable from CG150 and other Cs*Ab*. CG108 isolates displayed the lowest detectable levels of susceptibility across all beta-lactam antibiotics in KB-AST (Supplementary Figure 2). We observed comparably greater intra-CC variation in the susceptibility to other antibiotic classes, which strongly correlated with the presence of resistance elements repeatedly found in multiple lineages (Figure 3 and Supplementary Figure 2). The only major discrepancy was SXT nonsusceptibility in CG499 isolates who all lacked the *sul1/sul2* gene putatively conferring resistance in all other lineages, similar to CG499 isolates in prior studies [14].

### Atypical Resistance Phenotypes Displayed by CG108 and CG499 Isolates

Results from clinical laboratory MB-AST generally matched KB-AST for most isolates, but 2 consistent discrepancies were noted (Supplementary Data 1, 5). First, CG108 isolates displaying the lowest detectable susceptibility to SAM in KB-AST were reported as “intermediate” or “susceptible” according to MB-AST. Second, despite CG499 isolates lacking canonical resistance genes and being “susceptible” to SXT via MB-AST, they displayed decreased susceptibility to SXT in KB-AST. Comparable MB-AST results were observed when banked isolates were retested in our research laboratory (Supplementary Data 1). Closer review of KB-AST plates found that CG108 isolates against IPM and SAM and CG499 isolates against SXT each displayed subpopulations extending between their main lawn margin and the antibiotic disk borders (Figure 4*A*). Bacteria from the lawn or colonies at the antibiotic disk border were subcloned overnight without antibiotics, and secondary KB-AST was performed (Figure 4*B*). Each subclone pair yielded similar results in secondary KB-AST (Figure 4*C*-*D*). Similar SXT-related phenomenon was observed when testing unrelated ST499 isolates from St. Louis (Supplementary Figure 3) [14]. No other lineages displayed similar patterns in KB-AST.

### Comparative Analysis With *Ab* Isolates From Other U.S. Regions

The diversity of CR*Ab* lineages among isolates was unexpected, so we investigated how the UAB cohort related to the greater U.S. *Ab* population. We identified 545 genomes from *Ab* isolated in the United States after January 1 2010 (Supplementary Data 2) for intra-CG comparative analyses (Supplementary Table 2). UAB CG2 isolates occupied 3 branches within 2 previously described clades [9, 21], 1 branch in Clade C (isolates 021 and 187) and 2 in Clade A (isolate 028 and all the others, respectively) (Figure 5). Most CG1, CG499, CG108, and CG250 UAB isolates occupied exclusive branches within their lineages, though 2 CG250 (isolates 024 and 232) and CG108 isolates (isolates 076 and 260) were on distinct branches. Conversely, UAB CG150 and CG32 genomes were broadly distributed within their respective trees. No CG427 or CG164 isolates were identified among genomes from other regions, and 2 of the most common U.S. lineages, CG406 and CG79, were absent from the UAB cohort (Supplementary Table 2).

ARG analysis of the total cohort revealed intra-CG variability regarding strains harboring resistance genetic elements for non–β-lactam antibiotic classes (Supplementary Data 2). In contrast, the allelic identity of intrinsic OXA and ADC genes facilitated the delineation of phylogenetic subclades within CGs, most of which comprised isolates from different studies and regions (Supplementary Figure 4). Though OXA-23 was the most commonly encoded carbapenemase overall, OXA-24/72 carbapenemases were identified in CG2 subclades A2, C2, and G (Supplementary Figure 4B), half of CG79, 2 unrelated subclades in CG406, and nearly all non-Mountain region CG499 isolates (Supplementary Figure 4C). Notably, the FtsI A515 V polymorphism was encoded by isolates only in CG2 subclade B2 (Supplementary Figure 4B) and in CG108 subclades containing isolates lacking any carbapenemase (Supplementary Figure 4C). CG406 was the only CR*Ab-*associated lineage [8, 14] where most isolates lacked an identifiable carbapenem resistance element. No extrinsic β-lactamase genes were identified among CG150 and CG32 isolates (Supplementary Figure 4D).

## DISCUSSION

We report a comprehensive analysis of clinical cases associated with *Ab* isolates in a major U.S. Deep South healthcare center. With the aim of delineating the microbial landscape of *Ab* propagating in our region, we characterized the presentation of *Ab-*associated cases and the population structure of clinical isolates. Investigation of clinical outcomes and transmission dynamics is the subject of ongoing study, especially because many of the lineages identified in the region were absent in recent major studies [8].

Cs*Ab* cases are more prevalent than CR*Ab* in many U.S. regions [5, 22], but are often excluded from epidemiological surveys. We found that HA rates among CR*Ab* cases approximated those of Cs*Ab* cases in later years (Supplementary Data 3) and isolate tissue source distributions were indistinguishable (Figure 1*E*-*F*). However, consistent with reports from other regions [5, 23], seasonality only occurred among Cs*Ab* cases (Figure 1*A*-*B*). The Cs*Ab* and CR*Ab* population structures also differed: 37 unrelated lineages were identified among 47 Cs*Ab* isolates, but only 5 CGs were observed among 26 CR*Ab*. Two Cs*Ab* lineages (ie, CG150 and CG32) were associated with multiple independent cases in our cohort. However, these Cs*Ab* isolates did not appear to share recent common ancestors (Figure 5), and CG150 or CG32 isolates were only spuriously identified in other U.S. studies (Supplementary Figure 4D). Repeated observations of decreased relatedness among Cs*Ab* isolates [14] supports that Cs*Ab* disease is result of sporadic crossover events from environmental sources.

In contrast, we identified multiple CR*Ab* clonal clusters comprising isolates collected ≥90 days apart, from different tissue sources, and associated with HA and nHA cases (Figure 2). Though many of these isolates belong to subclades unique to our region, attributing CR*Ab* clusters to hyperlocalized outbreaks would require a formal clonality analysis beyond the scope of our current study. Regardless, these observations imply the existence of contemporary non-hospital CR*Ab* pools persisting in the region's patient population, as observed in other U.S. studies [9, 14].

We observed an unexpectedly high degree of CR*Ab* lineage heterogeneity of in our cohort. As an environmental species, *Ab* harbors a very diverse genetic pool even compared to other Gram-negative pathogens [24, 25], but recent studies have repeatedly shown only 1 or 2 lineages predominating in each healthcare center [13, 14]. It can be speculated that climate and/or socioeconomic factors in the U.S. Deep South are more permissive to *Ab* phylogenetic diversity than other regions [8, 12], akin to comparing *Ab* populations in Latin America to the rest of the world [8, 10]. Thus, we predict investigation of *Ab* in other Deep South regions will detect similar heterogeneity.

Historically, most CR*Ab* disease has been attributed to CG2 [8, 9, 13, 15], but new lineages have emerged across the United States in recent decades. CG499 was first described in 2008 (as a single ST123 isolate [11]) and 2010 (as a ST499 isolate [17]) but has now become the second most common CR*Ab* lineage in the United States and the dominant lineage in some centers [8, 12, 14]. CG108 was only sporadically observed in prior studies, but we demonstrate its capacity to establish a stable presence in a region. ST250 was first identified in a 2007 isolate from Puerto Rico [26], rarely identified in prior U.S. surveillance, but repeatedly isolated in our cohort. Last, though not identified in our cohort, CG406 has been exclusively identified in various U.S. regions in recent years [8, 14, 15]. Altogether, generalizing historical or even multicenter study findings to a region's *Ab* population structures should be avoided because the lineages responsible for local cases can differ drastically from site to site and over time.

The contribution of *Ab* phylogeny to clinical outcomes is unclear [3, 8]. We argue, however, that surveilling local CR*Ab* lineages’ unique resistance features can influence local medical practices. For example, as the most prevalent carbapenemase among CR*Ab*, OXA-23 is a target of current and emergent rapid molecular detection tools in clinical labs [27]. However, U.S. isolates belonging to CG2, CG108, and CG499 display carbapenem resistance via other genetic determinants (ie, OXA-24 or the FtsI A515 V polymorphism), and the mechanism of resistance in many CG406 isolates remains to be identified [8, 14, 16] (Supplementary Figure 4). Indeed, OXA-23 was detected in <50% of 6026 CR*Ab* isolates tested in the Antimicrobial Resistance Laboratory Network [28]. Last, as previously reported [14], we observed that the level of resistance conferred by a β-lactamase varies from lineage to lineage (ie, OXA-23-associated IPM resistance was greater among CG2 isolates compared to CG250 isolates) (Supplementary Figure 2). Altogether, the utility of OXA-23–based testing varies according to regional CR*Ab* population structures.

Another important finding was the discordance between MB-AST and KB-AST for SAM against CG108 isolates and for SXT against CG499 isolates. The repeated occurrence of subpopulations with different resistance levels and the instability of the increased resistance phenotype (Figure 4) are suggestive of phase-variable heteroresistance in these CGs. How these phenotypes are achieved is unclear, but the strict link between lineage and antibiotic class suggests involvement of separate mechanisms. This is especially true for CG499, which appears to achieve decrease susceptibility to SXT without any of the typical genetic elements associated with SXT resistance in other *Ab* lineages (Figure 3). Regardless, why these traits are not readily identifiable via MB-AST and how the spontaneous occurrence of increased SAM or SXT resistance influences clinical outcomes or susceptibility to new combination agents (ie, sulbactam-durlobactam) should be the focus of more immediate study.

Our work shows how knowledge of isolate lineage could influence clinical decision-making and sets a foundation to explore how lineage may influence other aspects of *Ab* disease, such as virulence, clinical prognosis, and propensity to be spread among certain patient populations. Though consistent with recent reports from other U.S. locations [12–14], our study was performed in a single healthcare center, and these early-stage findings encompass only a year's worth of isolates. Whether our findings are generalizable to nationwide trends or whether other centers in the U.S. Deep South harbor *Ab* populations with elevated degree of heterogeneity compared to other U.S. regions must be further investigated.

## Supplementary Data

Supplementary materials are available at *Open Forum Infectious Diseases* online. Consisting of data provided by the authors to benefit the reader, the posted materials are not copyedited and are the sole responsibility of the authors, so questions or comments should be addressed to the corresponding author.

## Notes

***Financial support*.** This work was supported by the National Institutes of Health (NIH), National Institute of Allergy and Infectious Diseases/grant number K08 AI148582, the University of Alabama at Birmingham IMPACT award, and a Pilot Grant from the Infectious Disease Clinical Research Consortium (funded by NIH 3UM1AI148684-04S2), all awarded to J.J.C.

***Patient consent statement.*** The requirement to obtain patient consent was waived for all protocols in this study, as approved by the University of Alabama at Birmingham (UAB) institutional review board (IRB# 30003572 and 30008212).

***Previous presentations.*** Preliminary results from this study were presented at the ASM Microbe conference; 15–19 June 2023, Houston, Texas, USA.

***Author Contributions.*** J.J.C. and E.G. conceptualized, designed and performed the study. S.M.L., D.B.M., and M.A.B contributed to design and completion of the study. E.G. wrote the manuscript first draft. All authors were involved with manuscript revisions and approved the final version.
