# Supplementary figures and images for: Epidemiological, Phylogenetic, and Resistance Heterogeneity Among Acinetobacter baumannii in a Large U.S. Deep South Healthcare system
Source: Open Forum Infect Dis. 2024 Aug 10;11(9):ofae458. doi: 10.1093/ofid/ofae458 (PMC11370794; doi:10.1093/ofid/ofae458)

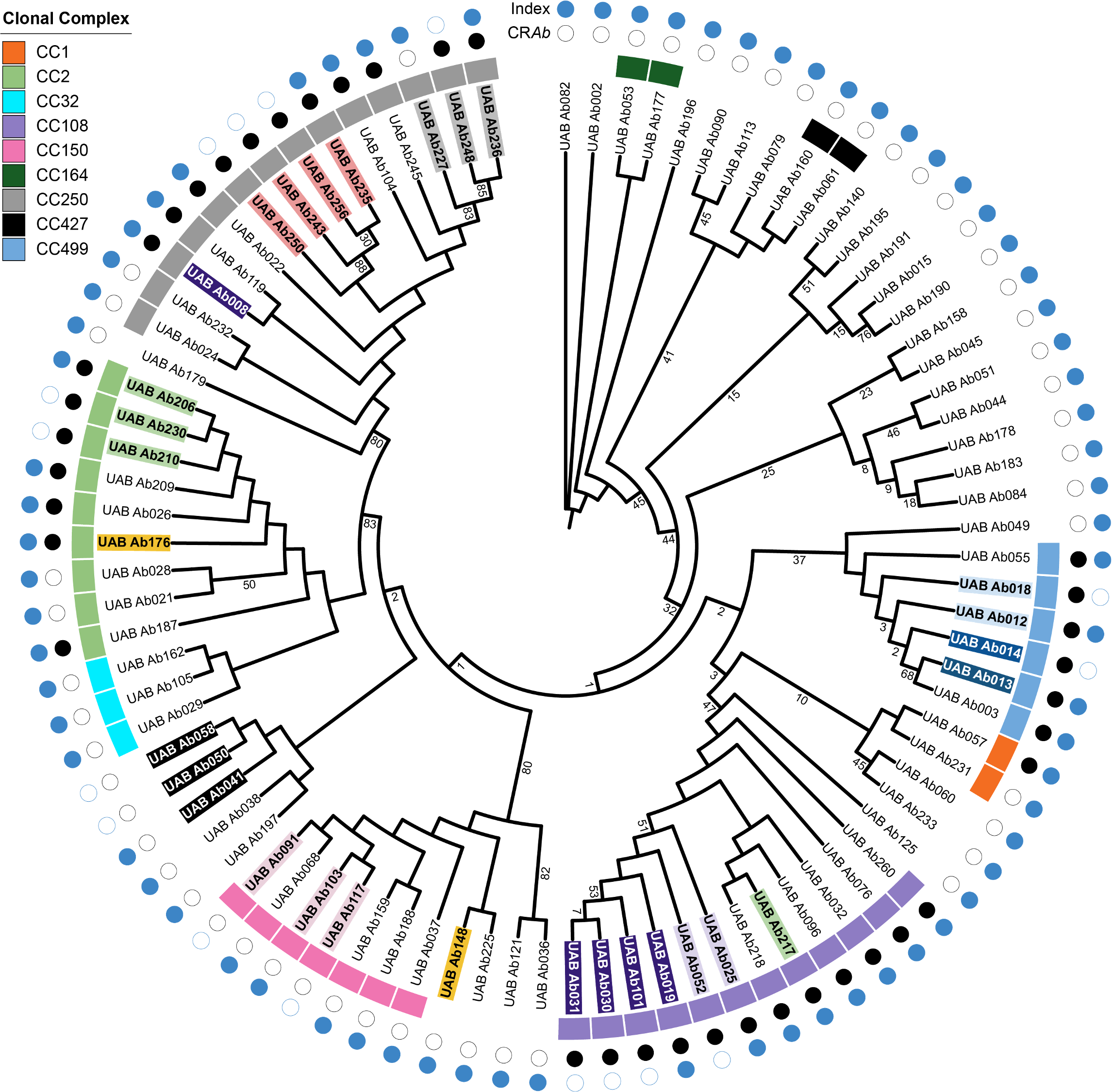

Supplement: ofae458_Supplementary_Data [file ofae458_supplementary_data.zip › FigureS1_UABall_phylo_tree.png]

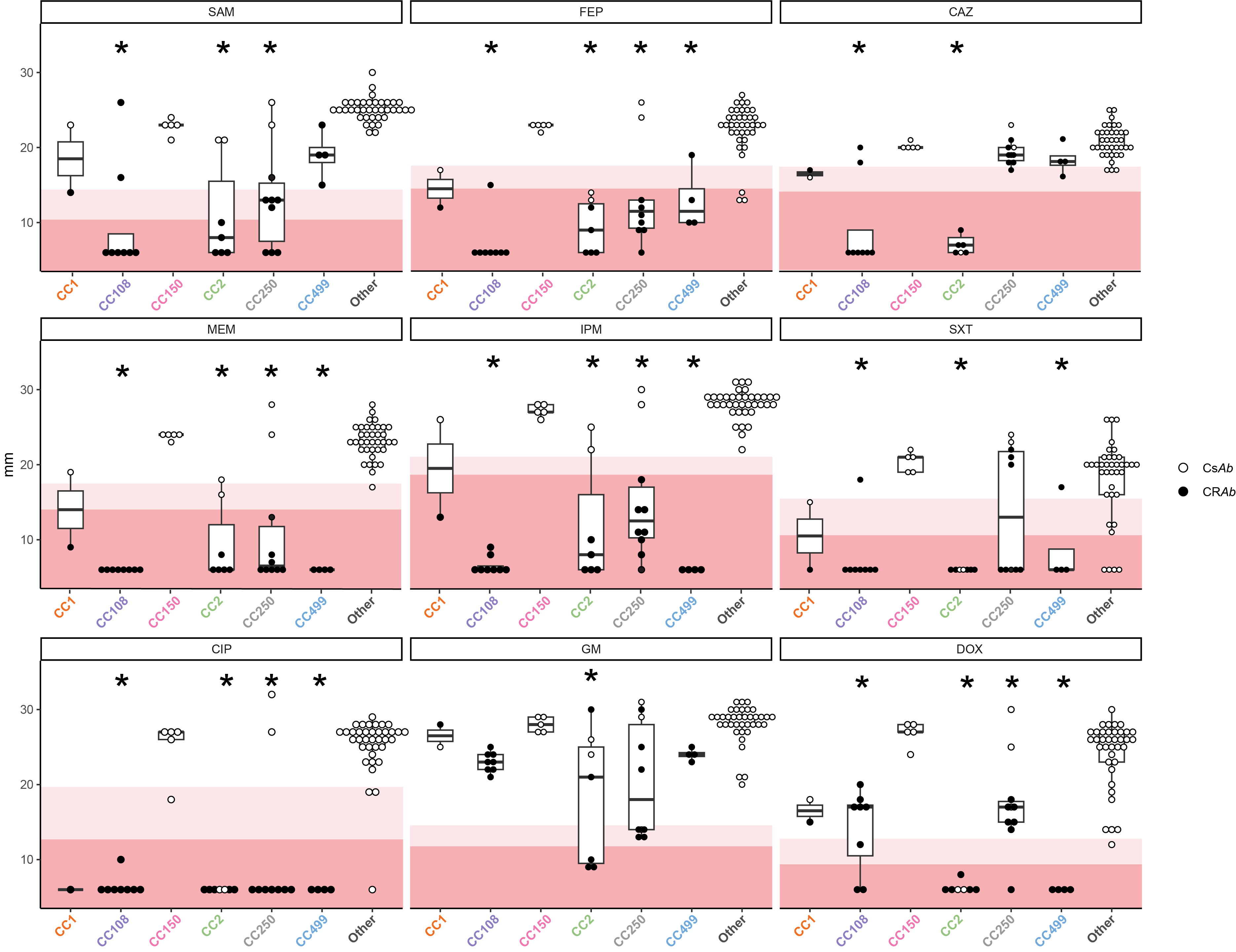

Supplement: ofae458_Supplementary_Data [file ofae458_supplementary_data.zip › FigureS2_AST.png]

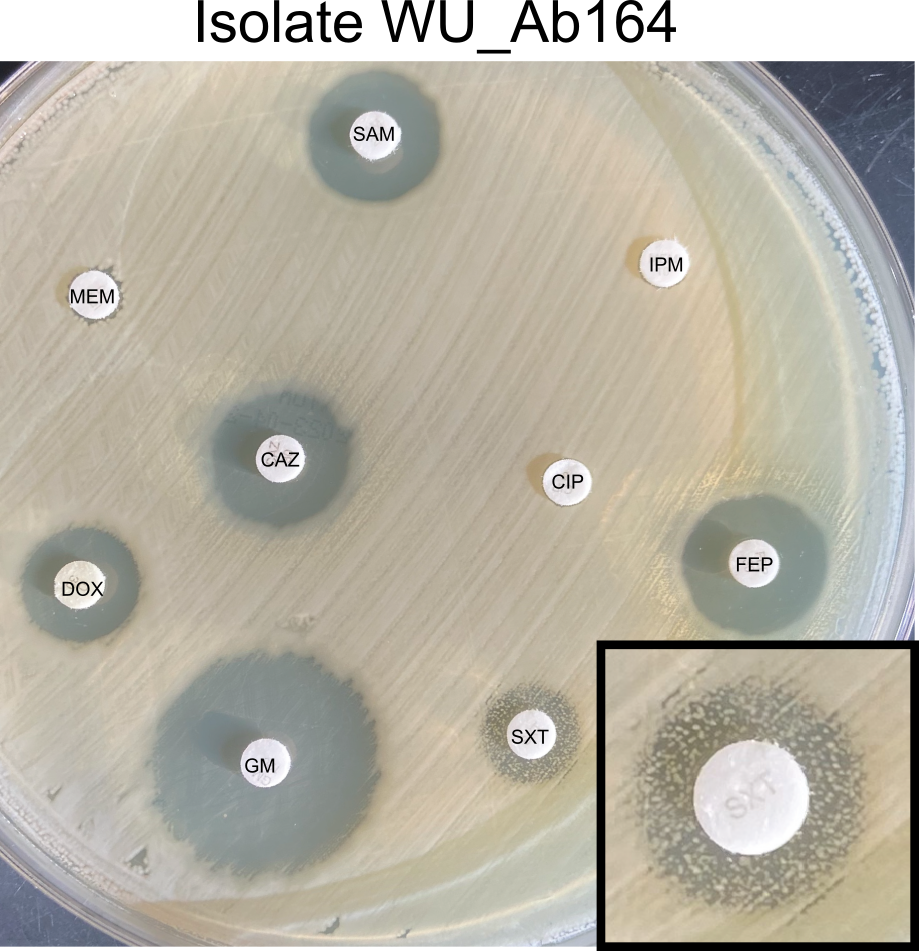

Supplement: ofae458_Supplementary_Data [file ofae458_supplementary_data.zip › FigureS3_WU164sxt.png]

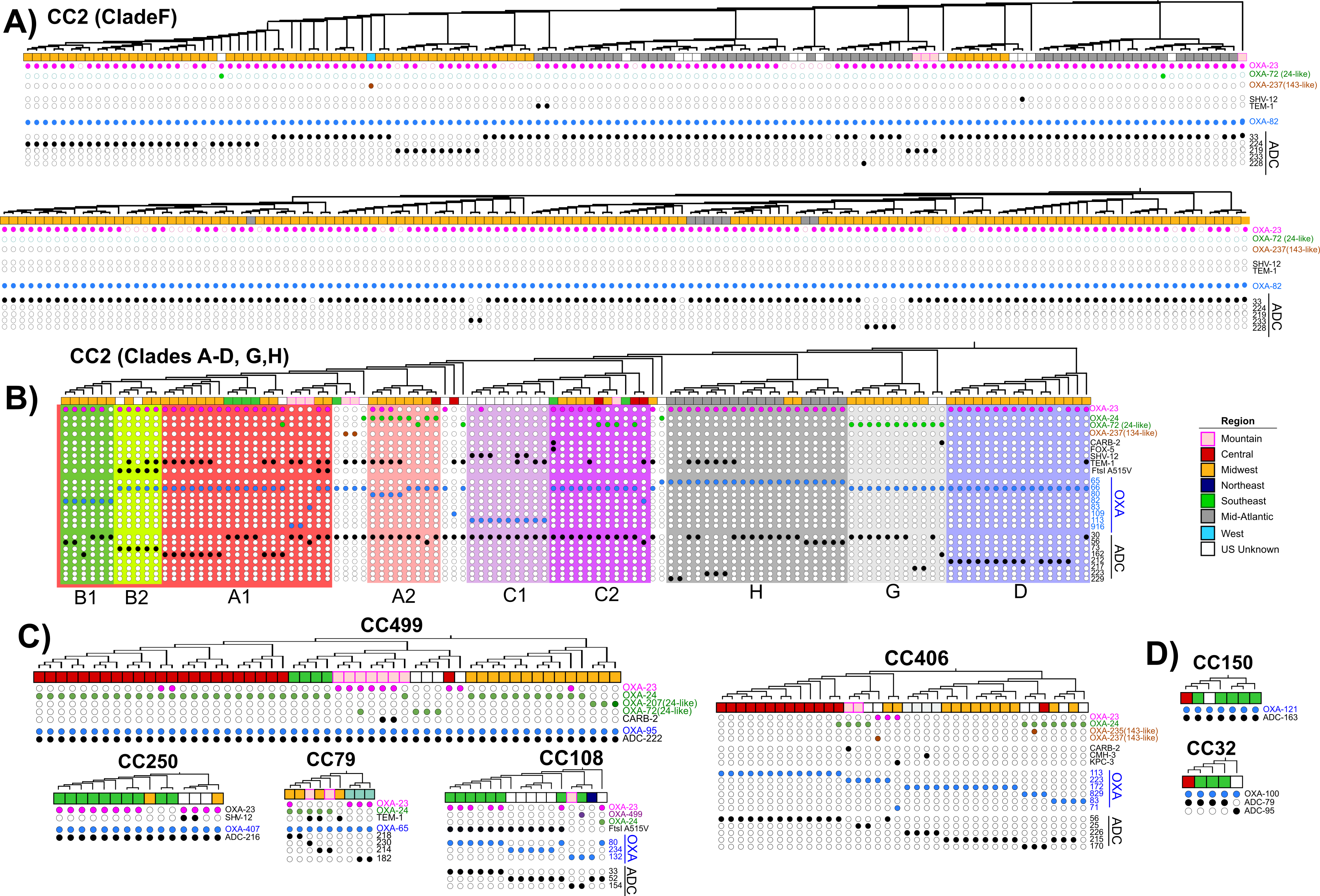

Supplement: ofae458_Supplementary_Data [file ofae458_supplementary_data.zip › FigureS4_US_CC_arg.png]

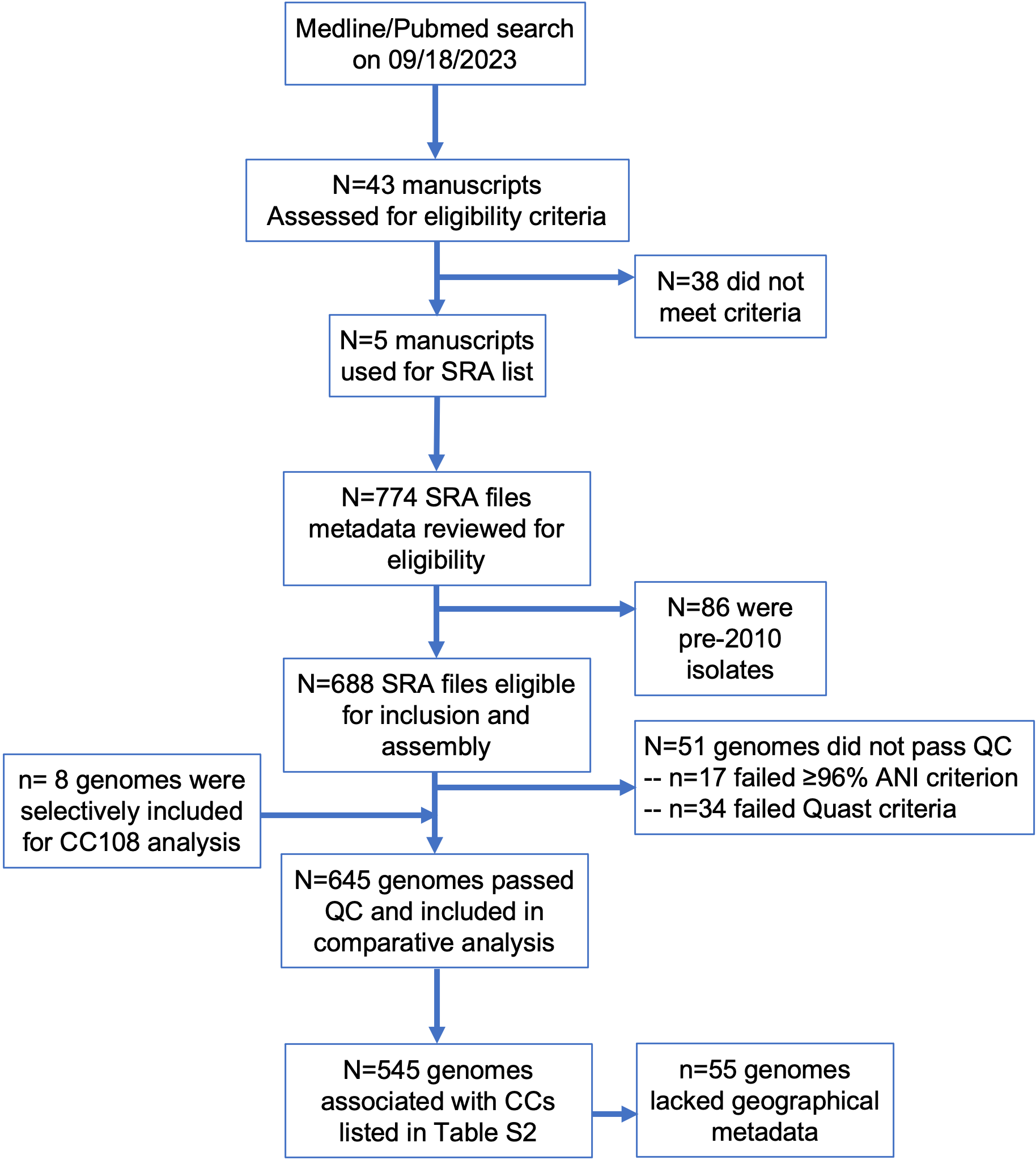

Supplement: ofae458_Supplementary_Data [file ofae458_supplementary_data.zip › FigureSuppData1.png]
