# Supplementary material for: Epidemiological, Phylogenetic, and Resistance Heterogeneity Among Acinetobacter baumannii in a Large U.S. Deep South Healthcare system
Source: Open Forum Infect Dis. 2024 Aug 10;11(9):ofae458. doi: 10.1093/ofid/ofae458 (PMC11370794; doi:10.1093/ofid/ofae458)
Supplement: ofae458_Supplementary_Data [file ofae458_supplementary_data.zip › 2023EG_MDCI1_supfigstables_v240718jjc.doc]

Supplementary figures and tables for

**Epidemiological, phylogenetic, and resistance heterogeneity among Acinetobacter baumannii in a large U.S. Deep South healthcare system**

Emma Graffice, Derek Moates, Sixto Leal, Megan Amerson-Brown, Juan J Calix^#^

^#^Corresponding author during review process. Email: [juancalix@uabmc.edu](mailto:juancalix@uabmc.edu)

**Figure S1: Phylogenetic clusters among all *Ab* sequenced isolates in this study.** Maximum likelihood tree of all UAB *Ab* isolates, rooted to midpoint. Branches with bootstrap values <90% are labeled. Highlighted and bolded leaves represent isolates obtained from the same patient, matched according to color. Outer boxes are color coded according to key to denote CGs containing >1 index isolate. Carbapenem resistance and index isolates are denoted by filled black and blue circles, respectively.

**Figure S2: Kirby-Bauer antimicrobial susceptibility testing (KB-AST) of UAB isolates.** Antibiotic susceptibility, according to zone of clearance (mm, y-axis), of CR*Ab* (black dots) and Cs*Ab* (white dots) index isolates grouped by CG. Backgrounds highlight ranges for “resistant” (dark) and “intermediate” (light) susceptibility, per CLSI guideline interpretation. Box-plot center lines denote medians; box limits, upper and lower quartile values and whiskers, 1.5 times the IQR. Medians were compared to CG150 using Mann-Whitney test with Bonferroni adjustment for multiple comparisons, with asterisks denoting P < 0.05. Abbreviations: CAZ, ceftazidime; CIP, ciprofloxacin; DOX, doxycycline; FEP, cefepime; GM, gentamicin; IPM, imipenem; MEM, meropenem; SAM, ampicillin-sulbactam; SXT, trimethoprim-sulfamethaxazole.

**Figure S3: CG499 isolates from an unrelated cohort also display variable SXT susceptibility.** Photos of KB-AST results of CG499 isolate WU_Ab164, against of all tested antibiotics and magnification of zone of clearance around SXT disk (inset). Abbreviations: CAZ, ceftazidime; CIP, ciprofloxacin; DOX, doxycycline; FEP, cefepime; GM, gentamicin; IPM, imipenem; MEM, meropenem; SAM, ampicillin-sulbactam; SXT, trimethoprim-sulfamethaxazole.

Table S1. Distribution of patient sex among UAB Ab cases, according to carbapenem susceptibility and isolate source.

Table S2: Clonal group breakdowns within our study and the global analysis.
