# Supplementary material for: Epidemiological, Phylogenetic, and Resistance Heterogeneity Among Acinetobacter baumannii in a Large U.S. Deep South Healthcare system
Source: Open Forum Infect Dis. 2024 Aug 10;11(9):ofae458. doi: 10.1093/ofid/ofae458 (PMC11370794; doi:10.1093/ofid/ofae458)
Supplement: ofae458_Supplementary_Data [file ofae458_supplementary_data.zip › 2023EG_UABMDCI_supmaterial_v6jjc.doc]

Supplementary Text for

**Epidemiological, phylogenetic, and resistance heterogeneity among *Acinetobacter baumannii* in a large U.S. Deep South healthcare systemouth healthcare system**

Emma Graffice, Derek Moates, Sixto Leal, Megan Amerson-Brown, Juan J Calix^#^

^#^Corresponding author during review process. Email: [juancalix@uabmc.edu](mailto:juancalix@uabmc.edu)

**Supplementary methods description**

**Methods used for whole genome sequencing and sequencing analysis.** Isolates were propagated on LB Miller agar, and genomic DNA was extracted with the Gentra Puregene Yeast/Bact. Kit (Qiagen, Germantown, MD, USA) according to manufacturer’s instructions. Library preparation and sequencing was performed at SeqCenter in Pittsburgh, PA using the Illumina NextSeq 2000 platform to obtain 2 x 150 bp reads. Following demultiplexing by barcode, reads had adapters removed with BBDuk v38.18[1]. Isolate sequencing raw reads or SRA files obtained from NCBI (see below) were assembled de novo using Unicycler[2] (v0.5.0), and contig .fasta files of assemblies that met the cutoff criteria of ≤200 contigs, genome length between 3.8 to 4.2 Mbp, and GC content between 38-40% according to QUAST[3] (v5.2.0), were included in downstream analysis. If genomes did not meet these criteria, they were removed from our study.

**Genomic analysis.** The species of each assembly was confirmed using the ANIm method from pyANI[4] (v0.2.12) with reference genomes of all *Acinetobacter* species. Average nucleotide identity of ≥96% compared to the *A. baumannii* reference genome (strain 19606, GCF_002811175.1) as the species cutoff[5]. Prokka[6] (v1.14.6) was used on contig files to identify open reading frames >500 bp in length and assigned gene names, if available. Antimicrobial resistance gene (ARG) content and multilocus sequence typing (MLST) were determined using AMRFinderPlus[7] (v3.11.4) and MLST[8] (v2.23.0) using the PubMLST database[9], respectively. Single locus variant sequence types (ST) with ≥4 genomes were grouped into CGs as listed in **Table S2**.

**Phylogenetic analysis.** We performed three levels of phylogenetic analysis on *Ab* genomes: Level 1) genomes of all UAB *Ab* isolates (n=89); Level 2) genomes of UAB index isolates (n=73); Level 3) UAB and NCBI-derived genomes grouped by clonal complex (CG). A CG was defined as a group of single locus variantSTs, according to Pasteur scheme of *Ab* MLST[9], and named according to the earliest ST assignment. Using .gff files produced by Prokka (above), Roary[10] (v3.13) was used to determine the core genes with the script “roary -e -n -p [number of threads] -i 95 *.gff”, we identified the core and accessory genomes (determined according to 95% nucleotide identity) for all levels. The resulting “core_gene_alignment.aln” file was used to generate a maximum likelihood tree with RaxML[11] (v8.2.12) for all levels with 100 iterations. All levels were visualized with iTOL[12]. Level 1 only included metadata from the isolate cases (Figure S1) and levels 2 and 3 metadata included visualization of ARG content and geographical region (Figures 3, 5, and S4).

**Molecular epidemiological systematic meta-analysis of genomes from recent U.S. surveys available on NCBI.** With the goal of comparing our local cohort with the contemporary nationwide *Ab* population structure, we systematically compiled genome sequences of *Ab* isolates obtained across the U.S. prior to 2010 (Figure Supp Materials). A PubMed/Medline search performed on September 18^th^, 2023 using different combinations of the search terms “*Acinetobacter baumannii,*” “epidemiology,” “whole genome sequencing,” and “United States” identified 48 peer-reviewed manuscripts. Manuscripts were manually reviewed by a researcher (EG) and were eligible for inclusion if they provided whole genome sequencing data of *Ab* clinical isolates collected after 2010 among patients in any U.S. region. Five studies met inclusion criteria (Table Supp Materials). Genomes included in these studies were extracted using provided NCBI SRA accession numbers (n=774) and assembled as described above. Genome metadata including Biosample accession, location of study, and year collected were compiled from NCBI where available. SRA from isolates obtained prior to 2010 were excluded. 637 of 774 (82%) SRA files yielded genome assembly .fasta files that passed QC and were incorporated into the comparative genomic analyses described above.

**Table. Studies included in systematic identification of U.S. *Ab* genomes.**

**Figure. Flow chart of process of how genomes were incorporated into molecular epidemiological metaanalysis.**

**Supplementary Text References**
